# Integrin-dependent cell adhesion to neutrophil extracellular traps through engagement of fibronectin in neutrophil-like cells

**DOI:** 10.1371/journal.pone.0171362

**Published:** 2017-02-06

**Authors:** Marcello Monti, Francesca Iommelli, Viviana De Rosa, Maria Vincenza Carriero, Roberta Miceli, Rosa Camerlingo, Giovanni Di Minno, Silvana Del Vecchio

**Affiliations:** 1 Dipartimento di Medicina Clinica e Chirurgia, Università degli Studi di Napoli “Federico II”, Naples, Italy; 2 Istituto di Biostrutture e Bioimmagini, Consiglio Nazionale delle Ricerche, Naples, Italy; 3 Dipartimento di Oncologia Sperimentale, IRCCS Istituto Nazionale Tumori “Fondazione G. Pascale”, Naples, Italy; 4 Dipartimento di Scienze Biomediche Avanzate, Università degli Studi di Napoli “Federico II”, Naples, Italy; Hospital for Sick Children, CANADA

## Abstract

Neutrophil extracellular traps (NETs), originally recognized as a host defense mechanism, were reported to promote thrombosis and metastatic dissemination of cancer cells. Here we tested the role of integrins α5β1 and ανβ3 in the adhesion of cancer cells to NETs. Neutrophil-like cells stimulated with calcium ionophore (A23187) were used as a stable source of cell-free NETs-enriched suspensions. Using NETs as an adhesion substrate, two human K562 cell lines, differentially expressing α5β1 and ανβ3 integrins, were subjected to adhesion assays in the presence or absence of DNAse 1, blocking antibodies against α5β1 or ανβ3, alone or in combination with DNAse 1, and Proteinase K. As expected DNAse 1 treatment strongly inhibited adhesion of both cell lines to NETs. An equivalent significant reduction of cell adhesion to NETs was obtained after treatment of cells with blocking antibodies against α5β1 or ανβ3 indicating that both integrins were able to mediate cell adhesion to NETs. Furthermore, the combination of DNAse 1 and anti-integrin antibody treatment almost completely blocked cell adhesion. Western blot analysis and immunoprecipitation experiments showed a dose-dependent increase of fibronectin levels in samples from stimulated neutrophil-like cells and a direct or indirect interaction of fibronectin with histone H3. Finally, co-immunolocalization studies with confocal microscopy showed that fibronectin and citrullinated histone H3 co-localize inside the web-structure of NETs. In conclusion, our study showed that α5β1 and ανβ3 integrins mediate cell adhesion to NETs by binding to their common substrate fibronectin. Therefore, in addition to mechanical trapping and aspecific adsorption of different cell types driven by DNA/histone complexes, NETs may provide specific binding sites for integrin-mediated cell adhesion of neutrophils, platelets, endothelial and cancer cells thus promoting intimate interactions among these cells.

## Introduction

Neutrophil extracellular traps (NETs) are web-like structures composed of nucleic acids, histones and selected cytoplasmic proteins that are released by activated neutrophils to entrap and kill different pathogens [1, 2]. In addition to their function as a host defense mechanism, a growing body of evidence indicates that NETs promote thrombosis by providing a scaffold for platelet and red blood cell adhesion [3, 4] as well as metastatic dissemination of cancer cells by entrapment of circulating tumor cells [5]. Furthermore, an increased number of peripheral blood neutrophils was found in tumor-bearing animals and these neutrophils were more prone to release NETs as compared to those derived from healthy animals providing consistent evidences of an association between NETs formation and cancer-associated thrombosis [6]. Moreover in a model of systemic infection, circulating tumor cells became trapped within NETs in lung capillaries [5]. Deposition of NETs within hepatic sinusoidal spaces was also associated with increased formation of hepatic micrometastases and subsequent development of gross metastatic lesions upon i.v. injection of cancer cells [5]. Although adhesion of cancer cells to neutrophil monolayer was enhanced by NETs release, the mechanisms by which NETs mediate adhesion and entrapment of circulating cancer cells have not been elucidated yet. A recent study in an animal model reproducing surgical stress of hepatic resection for metastatic colorectal cancer reported that NETs formation from mouse neutrophils was associated with High Mobility Group Box 1 (HMGB1) release and activation of Toll-like receptor 9 (TLR9)-dependent pathways in cancer cells promoting adhesion, proliferation, migration and invasion [7].

Based on these observations, we reasoned that members of integrin family, being the main mediators of cell adhesion, migration and invasion, may have a role in promoting cancer cell attachment to NETs. Integrins are heterodimeric membrane glycoproteins composed of non-covalently associated α and β subunits that bind to different components of the surrounding extracellular matrix [8]. Integrin ligation by its own natural ligands promotes intracellular signaling by co-clustering with kinases and adaptor proteins and by activating a number of intracellular mediators that ultimately lead to cell adhesion, migration, survival and invasion [9–11]. Integrins are expressed on the plasma membrane in an inactive status in which they do not bind their ligands and do not transduce signals unless exposed to activating external stimuli [12].

Human neutrophils express several integrins including those of the β1 and β2 subfamilies. The β1 family predominantly binds extracellular matrix (ECM) proteins such as collagen, fibronectin, and laminin mainly through the recognition of two amino acid sequences, namely Arg- Gly- Asp (RGD) and Leu-Asp-Val (LDV) [13]. The β2 integrins bind to complement, ICAM-1, fibrinogen, factor X and β-glucan [11, 12]. It is well established that neutrophils are recruited to tumor sites where they constitute a significant portion of inflammatory cell infiltrate and may have both pro and anti-tumoral properties [14].

Among members of the integrin family, ανβ3 has a prominent role in angiogenesis and metastatic dissemination. This integrin is indeed strongly upregulated at transcriptional level by pro-angiogenic growth factors or chemokines in activated endothelial cells. Expression and activation of this integrin was also found to be correlated with tumor invasion and metastases in a variety of human tumors [15]. In particular, activated ανβ3 is reported to cooperate with metalloproteinase and to strongly promote metastasis in human breast cancer cells [16–19]. Integrin ανβ3 is a receptor for RGD-containing proteins such as vitronectin, fibronectin and fibrinogen [8].

The aim of the present study was to test the role of integrins α5β1 and ανβ3 in the adhesion of cancer cells to NETs thus investigating the mechanisms underlying the interplay between cancer cells and neutrophils that may simultaneously promote a procoagulant state and metastatic dissemination.

## Materials and methods

### Cell lines and treatments

Human HL-60 cell line was kindly provided by Dr. M.P. Stoppelli [20] and was maintained in RPMI 1640 supplemented with 10% FBS. Then HL-60 cells were differentiated into neutrophil-like cells (dHL-60) by treatment with 1.3% DMSO in RPMI 1640 for 7 days [21, 22]. To confirm differentiation, changes in cell morphology were assessed by staining with May-Grunwald-Giemsa and the expression of CD11b, CD16b and CD177 antigens, markers of neutrophil differentiation, was evaluated by flow cytometry.

Human chronic myeloid leukemia K562 cells endogenously expressing α5β1 integrin (K562) and its derived clone, stably cotransfected with cDNA of αν and β3 subunits [23], overexpressing ανβ3 integrin (K562ανβ3), were a generous gift of Dr. S.D. Blystone. Cells were grown in Iscove's modified Dulbecco's medium (IMDM) (Gibco Life Technologies) containing 10% FBS [24]. Expression of α5β1 and ανβ3 integrins were tested by FACS analysis. All cells were maintained in a humidified incubator at 5% CO_2_ and 37°C.

To induce the release of NETs dHL-60 cells were treated with 2.5 and 25 μM calcium ionophore (A23187, Sigma-Aldrich) or vehicle. Briefly, differentiated cells were plated onto Petri dishes in RPMI 1640 with or without 10% FBS at a density of 1x10^6^ cells/ml and exposed to calcium ionophore for 4 h in a humidified incubator. After treatment, the conditioned medium was recovered and centrifuged at 310xg for 10 min at 4°C to obtain a cell-free NETs-enriched supernatant. This supernatant was then centrifuged at 18000xg for 10 min at 4°C and the pellet containing NETs was resuspended in 100 μl of cold PBS. Finally, double-stranded DNA concentration was determined using a NanoDrop ND-1000 spectrophotometer with V3.5.2 software (NanoDrop Technology, Cambridge, UK) and the NETs-enriched suspension was used as a stock for further experiments. Different stock suspensions were prepared and used to obtain experimental replicates.

### FACS analysis of integrin and antigen expression

Levels of CD11b, CD16b and CD177 antigens as well as expression of α5β1 and ανβ3 integrins were determined by fluorescence-activated cell sorting (BD FACSAria II) [24]. Briefly, dHL-60 cells (5-10x10^5^) were harvested and incubated with mouse monoclonal antibody anti-human CD11b conjugated with R-phycoerythrin (clone 2LPM19c, Dako), anti-human CD16b conjugated with APC (clone REA589, Miltenyi Biotec) or anti-human CD177 conjugated with FITC (clone REA258, Miltenyi Biotec) following manufacturers’ instructions for 1 h at 4°C in the dark. Then, cells were washed with PBS and analyzed by flow cytometry. Three independent experiments were performed for each antibody. Similarly, K562 and K562ανβ3 cells (5x10^5^) were incubated with 1 mg/ml mouse monoclonal antibodies HA5 and LM609 (Chemicon) recognizing α5β1 and ανβ3 integrins, respectively, for 1 h at 4°C in the dark. After washing with PBS, cells were incubated with FITC-conjugated anti-mouse IgG (Sigma-Aldrich), diluted 1:40, for 30 min at 4° C in the dark and then subjected to FACS using BD FACSDiva 8.0 software. Simultaneous representation of different histograms from the same cell line was obtained by Kaluza Flow Cytometry analysis V1.2 (Beckman Coulter).Two independent experiments were performed for each cell line and antibody.

### Qualitative and quantitative analysis of NETs

Fluorescence microscopy was used to evaluate NETs formation. Briefly, 5x10^5^ dHL-60 were seeded in 24 well tissue culture plates with glass coverslips in serum-free HBSS with calcium and magnesium chloride at 37°C with 5%CO_2_ and allowed to attach. Then cells were treated with calcium ionophore and stained with Sytox Green cell-impermeable nucleic acid dye (5 μM, Invitrogen). After washing with PBS, each coverslip was mounted on glass slide and examined by fluorescence microscopy.

Quantitative analysis of NETs formation was performed with Sytox Green followed by plate reader assay. Briefly, 1x10^5^ dHL-60 cells were seeded in 96-well black plate in 100 μl of serum-free HBSS with calcium and magnesium chloride and left for 1 h at 37°C, 5%CO_2_. After treatment with 2.5 and 25 μM A23187, Sytox Green was added at 5 μM for 10 min in a humidified incubator. The fluorescence was then measured using a PerkinElmer Multimode Plate Reader with EnSpire Manager Software and the results were expressed as percentage of total DNA release considering fluorescence readout obtained from cells lysed with 2% (vol/vol) Triton X-100 as 100% DNA release. Three independent experiments in triplicates were performed.

### Western blotting and immunoprecipitation

Since histone citrullination through the activation of peptidylarginine deiminase 4 (PAD4) is a necessary modification for NETs production [25, 26], levels of citrullinated histone H3 (Cit H3) were assayed in samples of conditioned media and NETs stocks. Protein content of each sample was evaluated by Bradford assay after DNAse 1 treatment (40 UI/ml, Roche) for 15 min at room temperature followed by centrifugation at 400xg for 5 min at 4°C. Protein samples (50 μg, if not differently indicated) were subjected to SDS PAGE and western blot analysis using standard protocols. Primary antibodies used for western blotting included anti-citrullinated histone H3 (citrulline R2+R8+R17) (Abcam), total anti-histone H3, anti-α5 chain (Chemicon), anti-β1 chain (Chemicon), anti-β3 chain (Santa Cruz) rabbit polyclonal antibodies as well as anti-fibronectin (clone 10/fibronectin, BD Biosciences), anti-vitronectin (clone VIT-2, Sigma) and anti-αv chain (clone P2W7, Santa Cruz) mouse monoclonal antibodies. A commercially available ECL kit (GE Healthcare) was used to reveal the reaction. Gels were stained with coomassie dye R-250 using Imperial^TM^ protein stain (Thermo Fisher Scientific) to obtain SDS-PAGE patterns of protein content and ensure equal loading.

Proteins (1.5 mg) from conditioned media of unstimulated and A23187-stimulated neutrophil-like dHL-60 cells in RPMI 1640 with 10% FBS were incubated with 2.5 μg/ml of anti-fibronectin mouse monoclonal antibody overnight at 4°C under gentle rotation. The immunoprecipitated proteins recovered by EZview Red Protein A Affinity Gel (Sigma-Aldrich) beads were separated by SDS-PAGE and analyzed by immunoblotting using the indicated antibodies.

### Cell adhesion assays to NETs

Human K562 cells differentially expressing α5β1 and ανβ3 integrins were subjected to adhesion assays using NETs as an adhesion substrate. Briefly, 24-well flat-bottom plates were incubated overnight at 4°C with 5 μg of NETs stock in 200 μl of PBS and with diluent or conditioned medium from unstimulated dHL-60 cells as negative controls. After gentle washing with PBS and incubation with serum-free IMDM with 1% BSA for 1 h at room temperature, K562 (1.5x10^5^ cells/well) and K562ανβ3 (3x10^5^ cells/well) cells were added and allowed to adhere for 1h at 37°C. Nonadherent cells were then removed by washing each well with IMDM whereas adherent cells were trypsinized and counted. Data are expressed as percentage of adherent cells compared to the total number of added cells. As negative controls, cells were seeded onto NETs coated wells that had been pre-treated with DNAse 1 (10000 UI/ml, Roche) for 15 min at room temperature to induce optimal degradation of NETs as reported by other authors [5, 27, 28] and in S1 Supporting Information and S1 Fig. To evaluate the role of the selected integrins in promoting cell adhesion to NETs, cells were pre-treated with 45 μg/ml for 1 h at 4°C with anti-α5β1 P1D6 (Abcam) or anti-ανβ3 LM609 mouse monoclonal blocking antibodies and then seeded onto NETs coated plates at the final antibody concentration of 15 μg/ml. To test the adhesion properties of protein components of NETs, Proteinase K (1.8 mg/ml, Invitrogen) was added to pre-coated wells for 30 min at 37°C and then removed. Three independent adhesion assays, including all experimental conditions, were performed using different NETs stocks obtained from the stimulation of newly differentiated HL-60 cells in the presence of 10% FBS. Three additional adhesion assays were performed using NETs released from neutrophil-like cells stimulated in the absence of serum, i.e. in exogenous vitronectin and fibronectin-free conditions.

### Co-immunolocalization studies by confocal microscopy

Co-immunolocalization studies were performed by 510 META LSM confocal microscopy (Carl Zeiss). Briefly, neutrophil-like cells (2x10^5^) were seeded in serum-free HBSS and allowed to attach onto glass coverslip in 24-well flat-bottom plates for 1 h at 37°C. Then cells were treated with calcium ionophore A23187 (25 μM) to induce NETs release. Cells were fixed in formalin (2.5%) for 10 min at 4°C and incubated with rabbit polyclonal anti-citrullinated histone H3 (10 μg/ml) (Abcam) and mouse monoclonal anti-fibronectin (2.5 μg/ml) (clone 10/fibronectin, BD Biosciences) antibodies alone or in combination for 1 h at room temperature in the dark. Parallel experiments were performed by incubating permeabilized cells (0.1% Triton X-100) with mouse monoclonal antibody recognizing myeloperoxidase (MPO) (10 μg/ml, clone 2C7, Abcam). In an additional set of experiments glass coverslips were incubated overnight at 4°C with 5 μg of cell-free NETs enriched suspension and then subjected to co-immunolocalization study using anti-Cit H3, anti-MPO and anti-fibronectin antibodies as described. After washing with PBS containing 1% BSA, 1:700 goat Alexa Fluor 594 anti-rabbit IgG and 1:500 rabbit Alexa Fluor 488 anti-mouse IgG (Molecular Probes) were added for 45 min at room temperature in the dark. Then glass coverslips were washed twice with PBS, mounted with ProLong Gold Antifade Reagent (Invitrogen) and examined by confocal microscopy.

### Statistical analysis

Statistical analysis was performed using the software MedCalc for Windows, version 10.3.2.0, (MedCalc Software, Mariakerke, Belgium). Data are expressed as mean ± SD if not differently indicated. Unpaired Student’s t test was used to compare means. A p value < 0.05 was considered statistically significant.

## Results

**Fig 1:**
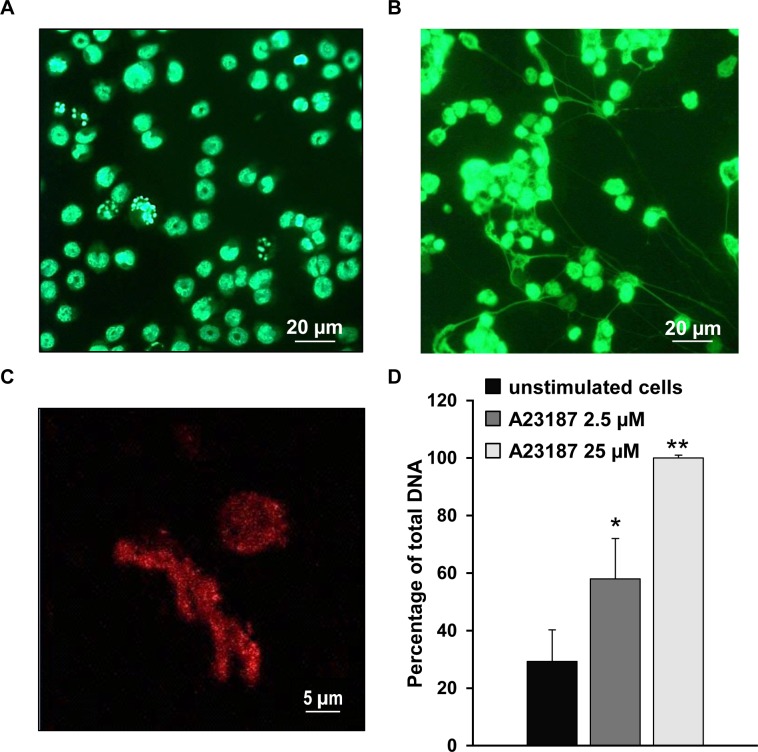
Qualitative and quantitative analysis of NETs formation induced by treatment of neutrophil-like cells with A23187. (A and B) Representative images obtained by fluorescence microscopy of neutrophil-like cells stained with Sytox Green cell-impermeable nucleic acid dye in basal conditions (A) and after stimulation with 25 μM A23187 for 4 h. Scale bar, 20 μm (B). Representative image obtained by fluorescence microscopy at higher magnification showing morphological details of NETs stained with antibody recognizing Cit H3. Scale bar, 5 μm (C). Results of three independent fluorescence plate reader assays in unstimulated and stimulated neutrophil-like cells expressed as percentage of total DNA released from lysed cells (D).

HL-60 cells were differentiated in neutrophil-like cells by treatment with DMSO for seven days. The efficiency of differentiation was tested by determining the expression of CD11b, CD16b and CD177 antigens and the mean percentages of positively stained cells were 60% ± 16%, 84% ± 19% and 78% ± 16%, respectively. Neutrophil-like cells were then stimulated to release NETs using calcium ionophore and NETs formation was qualitatively and quantitatively evaluated. Fig 1 shows representative images of NETs stained with Sytox Green and analyzed by fluorescence microscopy. Positively stained chromatin structures were found in the extracellular space of stimulated neutrophil-like cells (Fig 1B) whereas they were absent in untreated differentiated cells (Fig 1A). Furthermore, NETs and stimulated cells were positively stained for citrullinated histone H3 (Cit H3), a recognized marker of NETs structure (Fig 1C) [25, 26]. Quantitative analysis of NETs formation was performed by Sytox green plate reader assay. Fig 1D shows a dose-dependent response of NETs formation induced by increasing concentration of calcium ionophore. Considering fluorescence readout obtained from lysed cells as 100%, the percentage of extracellular DNA released by untreated cells was 29% ± 11% whereas treatment of dHL-60 cells with 2.5 μM and 25 μM of calcium ionophore caused 58% ± 14% and 100% ± 1% DNA release, respectively.

**Fig 2:**
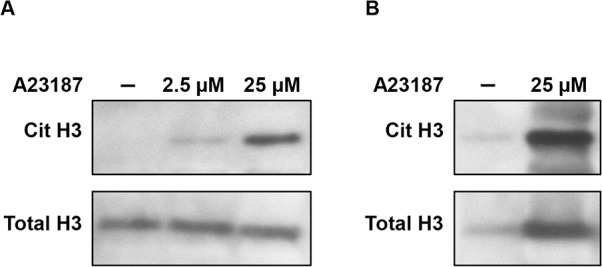
Levels of total and citrullinated histone H3 in NETs. Western blot analysis of total and citrullinated histone H3 (Cit H3) in conditioned media (A) and in NETs-enriched suspension (B) from stimulated neutrophil-like cells compared to the corresponding negative control.

To confirm that released NETs contained Cit H3, western blot analysis was performed on samples of conditioned media from neutrophil-like cells treated or not with increasing concentrations of calcium ionophore and on samples from NETs stock. Fig 2A shows undetectable levels of Cit H3 in the conditioned medium of untreated cells whereas a dose-dependent increase of this marker was observed in response to calcium ionophore. As expected, even higher levels of Cit H3 were found in samples of NETs-enriched suspensions obtained by centrifugation of conditioned media from stimulated cells whereas the protein was faintly detected in the corresponding samples from unstimulated cells (Fig 2B).

**Fig 3:**
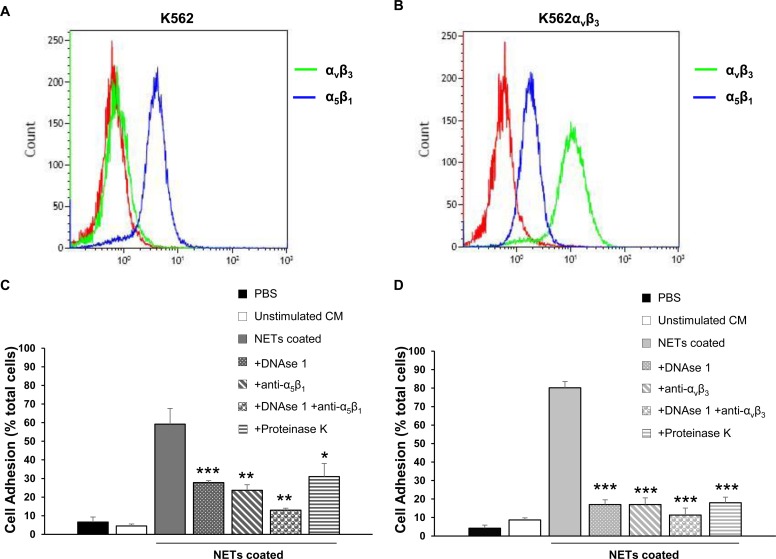
Integrin expression and cell adhesion to NETs. (A and B) Relative levels of α5β1 and ανβ3 integrins in human K562 cells (A) and its derived clone K562ανβ3 (B) were determined by flow cytometry analysis using primary monoclonal antibodies HA5 (blue histograms) and LM609 (green histograms) recognizing α5β1 and ανβ3 integrins, respectively, and compared to the corresponding negative control (red histograms). Two independent experiments were performed for each cell line and antibody. (C and D) Cell adhesion assays to NETs in K562 (C) and K562ανβ3 (D) cells. Three independent experiments were performed using different NETs stocks obtained from the stimulation of newly differentiated HL-60 cells in the presence of 10% FBS. Results are expressed as percentage of adherent cells compared to the total number of added cells and mean ± SD of three independent experiments is reported for each condition. Statistical significant differences between NETs coated and each experimental condition is indicated by symbols *, ** and ***, meaning p<0.05, p< 0.01 and p<0.001, respectively.

To test whether expression of integrins α5β1 and ανβ3 may modulate cell adhesion to NETs, K562 and K562ανβ3 cells differentially expressing the two integrins were selected for cell adhesion assays to NETs. We preliminarily tested the expression of the two integrins in each cell line by flow cytometry and their relative levels are shown in Fig 3A and 3B. As expected, K562 cells endogenously expressed α5β1 integrin whereas levels of ανβ3 were undetectable in this cell line showing a fluorescence intensity similar to that of negative control. Conversely, K562ανβ3 cells expressed higher levels of ανβ3 integrin as compared to α5β1 antigen. Although the single chains α5 and β1 were equally expressed in K562 and K562ανβ3 cells as shown by western blot analysis (S2 Fig) and as reported by other authors [29, 30], the whole α5β1 integrin was detected in 75% ± 19% of K562 cells and in 10% ± 10% of K562ανβ3 cells by FACS analysis using an antibody recognizing the whole integrin (S3 Fig). This apparent discrepancy may be explained by the impaired assembly of α5β1 integrin on the plasma membrane of K562αvβ3 due to the predominant expression of αv and β3 chains in this cell line. The whole ανβ3 integrin was indeed expressed in 94% ± 1% of K562ανβ3. Cell adhesion assays on NETs coated plates were then performed in the presence or absence of DNAse 1, blocking antibodies against α5β1 or ανβ3, alone or in combination with DNAse 1, and Proteinase K. Fig 3C and 3D show the results of the adhesion assays expressed as percentage of adherent cells over the total number of seeded cells. When K562 cells were added to NETs coated plates, the mean percentage of adherent cells was 59% ± 8%. A statistically significant decrease of the percentage of adherent cells was observed after the addition of DNAse 1 (28% ± 1%, p<0.0001) or anti-α5β1 antibody (24% ± 3%, p<0.001) indicating that both nucleic acid and an integrin substrate were critical for cell adhesion to NETs. Interestingly the combination of DNAse 1 and anti-α5β1 antibody almost completely blocked cell adhesion showing a mean percentage of adherent cells of 13% ± 1% (p = 0.0001). This value was significantly lower than that obtained with each agent alone (vs DNAse, p<0.0001; vs anti-α5β1, p = 0.0045). Negative controls performed with PBS or conditioned medium of unstimulated neutrophil-like cells showed a mean cell adhesion of 7% ± 3% and 5% ± 1%, respectively. Similarly, cell adhesion assays of K562ανβ3 cells to NETs coated plates showed 80% ± 3% of adherent cells that significantly decreased to 17% ± 3% in the presence of DNAse 1 (p<0.0001) and to 17% ± 4% when anti-ανβ3 blocking antibody was added (p<0.0001). The combination of DNAse 1 and anti-ανβ3 antibody resulted in 11% ± 4% of adherent cells, a value lower than that observed with DNAse 1 (p<0.05) and anti-ανβ3 antibody (p = 0.1017) alone. Negative controls performed with PBS and conditioned medium of unstimulated neutrophil-like cells showed a mean cell adhesion of 4% ± 2% and 9% ± 1%, respectively. In both K562 and K562ανβ3 cells, the addition of Proteinase K caused inhibition of cell adhesion (p<0.05 for K562 and p<0.001 for K562ανβ3) confirming the crucial role of the protein content of NETs structure in cell adhesion. Similar results were obtained using NETs released from neutrophil-like cells stimulated in serum-free conditions, i.e. in exogenous vitronectin and fibronectin-free conditions (Table 1). In particular, a significant reduction of cell adhesion was observed after treatment with DNAse 1 or anti-integrin antibody in both K562 (DNAse 1, p<0.01; anti-α5β1, p<0.01) and K562ανβ3 (DNAse 1, p<0.0001, anti-ανβ3, p<0.0001) cell lines.

**Table 1:** Adhesion of K562 and K562ανβ3 cells to NETs.

	K562 cells	K562ανβ3 cells
PBS	11% ± 7%	6% ± 1%
Unstimulated CM	7% ± 4%	4% ± 2%
NETs coated	62% ± 14%	61% ± 1%
+ DNAse 1	10% ± 7%	10% ± 2%
+ anti-integrin blocking Ab	10% ± 3%	8% ± 3%
+ DNAse 1 + anti-integrin blocking Ab	6% ± 3%	5% ± 2%
+ Proteinase K	21% ± 4%	19% ± 4%

Adhesion of K562 and K562ανβ3 cells to NETs obtained from neutrophil-like cells stimulated with 25 μM A23187 in serum free conditions. Data are expressed as percentage of adherent cells compared to the total number of added cells and the mean ± SD of three independent experiments is reported for each condition.

**Fig 4:**
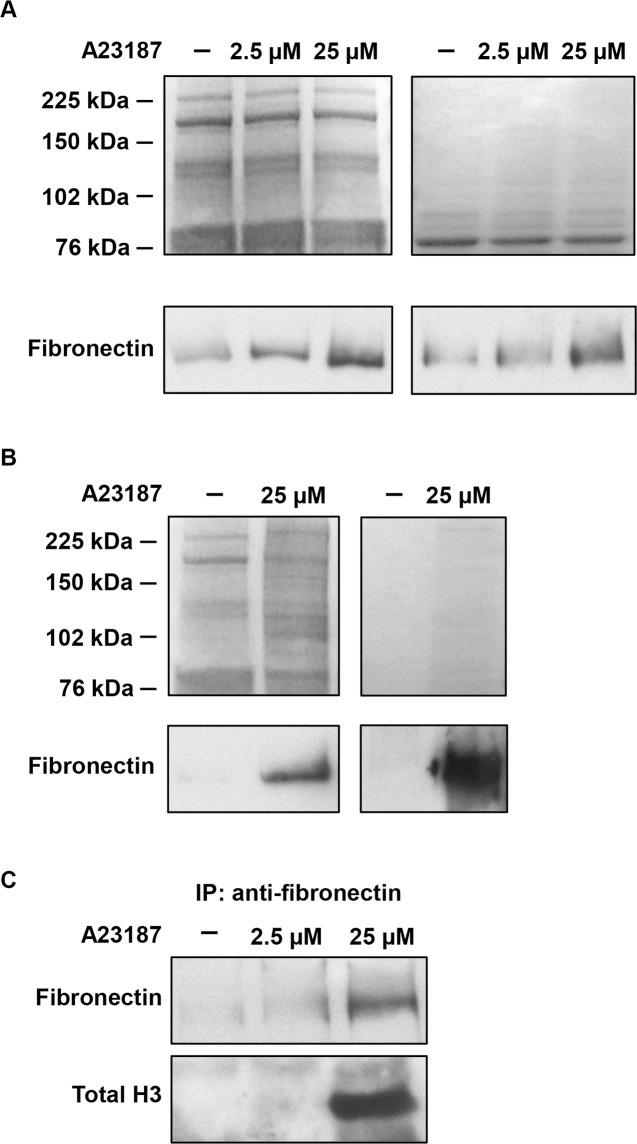
Levels of fibronectin and interaction with histone H3 in NETs. (A) Levels of fibronectin were determined by Western blot analysis performed on samples of conditioned media of neutrophil-like cells stimulated or not with increasing concentration of A23187 in the presence (left, 50 μg of protein per lane) or absence (right, 5 μg of protein per lane) of serum. (B) Western blot analysis of samples of NETs preparations obtained by stimulating neutrophil-like cells with 25 μM A23187 for 4 h in the presence (left, 50 μg of protein per lane) or absence (right, 5 μg of protein per lane) of serum compared to the corresponding negative control from unstimulated cells. Gels were stained with Imperial protein stain to obtain SDS-PAGE patterns of protein content and ensure equal loading. (C) Immunoprecipitation of conditioned media of unstimulated and A23187-stimulated neutrophil-like cells was performed using anti-fibronectin antibody followed by SDS-PAGE of the immunoprecipitated proteins and immunoblotting with the indicated antibodies.

Since both α5β1 and ανβ3 integrins bind with high affinity to fibronectin and vitronectin containing the RGD amino acid sequence, we tested whether NETs-enriched suspensions and conditioned media of stimulated and unstimulated neutrophil-like cells contained these integrin substrates. Western blot analysis showed a dose-dependent increase of fibronectin levels in the conditioned medium of stimulated neutrophil-like cells whereas a faint fibronectin signal was observed in samples of conditioned medium from unstimulated cells using both standard and serum free conditions (i.e. in exogenous vitronectin and fibronectin-free conditions) (Fig 4A). High levels of fibronectin were also found in samples of NETs-enriched suspensions obtained by stimulating neutrophil-like cells in both standard and serum-free conditions whereas the protein was undetectable in the corresponding negative control (Fig 4B). Vitronectin levels were undetectable in all samples (S4 Fig).

Immunoprecipitation of proteins from conditioned media of unstimulated and stimulated neutrophil-like cells showed an undetectable, faint or strong signal for fibronectin in samples from untreated, 2.5 μM and 25 μM A23187 treated cells, respectively (Fig 4C). Furthermore a strong signal for histone H3 was found only in conditioned medium of highly stimulated neutrophil-like cells indicating that fibronectin directly or indirectly interacts with histone H3.

**Fig 5:**
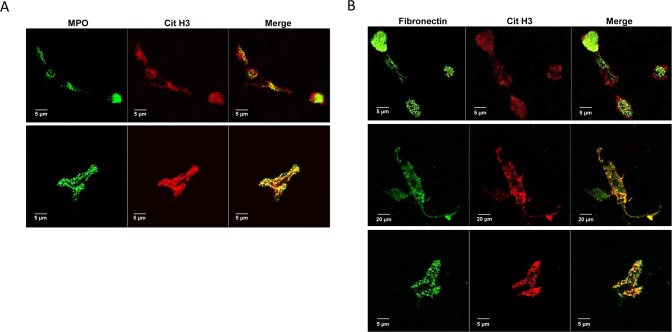
Co-localization of citrullinated histone H3 with myeloperoxidase and fibronectin in NETs. (A) Representative images obtained with confocal microscopy showing co-localization of MPO (green) and Cit H3 (red) in neutrophil-like cells stimulated in serum-free conditions with 25 μM A23187 for 4 h (upper panels) or in cell-free NETs enriched suspension (lower panels). Merged images showed the co-localization of MPO and Cit H3 confirming the typical features of NETs. (B) Representative images obtained with confocal microscopy showing co-localization of fibronectin (green) and Cit H3 (red) in neutrophil-like cells stimulated in serum-free conditions with 25 μM A23187 for 4 h (upper and middle panels) or in cell-free NETs enriched suspension (lower panels). Merged images showed a clear co-localization of Cit H3 and fibronectin in the structure of NETs at early (upper panels) and late (middle panels) phases of the process coexisting in different fields as well as in NETs after their isolation procedures (lower panels).

To confirm the typical features of NETs, co-immunolocalization studies of Cit H3 (red) and MPO (green) were performed using confocal microscopy in A23187-stimulated dHL-60 cells and in isolated NETs (Fig 5A). Fusion images in Fig 5A showed that Cit H3 and MPO co-localize within NETs structure. To test whether fibronectin is localized within NETs, co-immunolocalization studies of fibronectin and Cit H3 were performed using confocal microscopy in stimulated neutrophil-like cells and in NETs stock (Fig 5B). Representative images of NETs in the extracellular space of stimulated neutrophil-like cells (Fig 5B, upper and lower panels) positively stained with anti-fibronectin antibody (green) and with anti-Cit H3 antibody (red) and merged images showed a clear co-localization of the two proteins in the structure of NETs. Similar results were obtained in cell-free NETs enriched suspension (Fig 5B, lower panels). These findings taken together confirmed that fibronectin is localized within NETs and modulates cell adhesion to NETs through the engagement of α5β1 and ανβ3 integrins, providing mechanistic clues on the in vivo interaction of NETs with different types of cells expressing these integrins including peripheral blood or activated endothelial cells as well as cancer cells.

## Discussion

Our study showed that α5β1 and ανβ3 integrins mediate cell adhesion to NETs by binding to their common substrate fibronectin, which was found to co-localize with Cit H3 inside the web-like structure of NETs and to interact directly or indirectly with histone H3. Treatment with DNAse 1 and blocking antibodies against α5β1 and ανβ3 integrins inhibited cell adhesion to NETs and when used in combination almost completely blocked cell adhesion indicating that both DNA and fibronectin were relevant in determining cell attachment. From quantitative data of cell adhesion, it is conceivable that treatment with DNAse 1 alone would digest DNA, disrupt the web-like structure of NETs and prevent the interaction of DNA/histone complexes with fibronectin thus inhibiting cell adhesion not only to DNA but also to fibronectin. Since treatment with blocking anti-integrin antibodies resulted in a reduction of cell adhesion similar to that obtained with DNAse 1, it is likely that cell adhesion to NETs may start with integrin-binding to fibronectin that would attract cells near to DNA/histone complexes allowing a stable cell interaction with DNA.

NETs incubated with plasma were reported to bind to several plasma proteins including fibronectin, von Willebrand factor and fibrinogen [3]. In our experiments, fibronectin was originated from neutrophil-like cells, since its levels were faintly detected in the conditioned medium of unstimulated neutrophil-like cells and increased in a dose-dependent manner in response to A23187. It is unclear whether fibronectin enter the structure of NETs during their formation or simply binds to NETs in the extracellular space. In this respect fibronectin was reported to have a moderately high affinity for eukaryote double-stranded DNA and a DNA-binding domain was described in human plasma fibronectin [31]. Another well-known DNA binding protein, HMGB1, was reported to be a component of NETs and increased levels of HMGB1 were found in the conditioned medium of stimulated neutrophils [7]. Furthermore HMGB1-dependent activation of TLR9 pathways was found to occur in cancer cells upon exposure to NETs and to increase proliferation, invasion and migration [7]. Our findings support these observations since specific fibronectin domains are reported to be agonists of TLRs [32] and HMGB1/TLR or HMGB1/RAGE axes directly or indirectly modulate fibronectin expression and fibronectin-dependent migration [33].

Two distinct pathways of NETs formation have been reported in human neutrophils: the PMA-induced NOX-dependent [2, 34] and the calcium ionophore-induced NOX-independent mechanisms [26]. The end point of both mechanisms is chromatin decondensation associated with histone citrullination followed by extrusion of nuclear DNA into the extracellular environment. The process is dependent on the generation of reactive oxygen species (ROS) and the migration of the protease neutrophil elastase (NE) and myeloperoxidase (MPO) from granules to the nucleus. The disruption of nuclear membrane that occurs during the process leads to the coalescence of nucleoplasm and cytoplasm so that cytoplasmic proteins including fibronectin can bind to DNA/histone complexes.

Cellular fibronectin is synthesized by many cell types, including fibroblasts and endothelial cells. It is an old notion that neutrophils, in addition to carry receptors for fibronectin on their plasma membrane, are also able to synthesize and secrete fibronectin [35, 36] especially at inflammatory and tissue injury sites. Similarly, HL-60 cells are reported to secrete fibronectin and to acquire receptors for fibronectin during their differentiation along the granulocytic pathway [37]. The main implication of the presence of fibronectin in the web-like structure of NETs is that it provides specific binding sites for several integrins expressed on the plasma membrane of neutrophils, platelets, endothelial and cancer cells. Therefore, in addition to mechanical trapping and aspecific adsorption of different cell types driven by DNA/histone complexes, integrin-mediated cell adhesion to NETs should be taken into account as a mechanism promoting cell-cell interactions at the interface with NETs. By preventing fibronectin binding to integrins, specific inhibitors or antibodies may disrupt such cell-cell interactions and impair homing of circulating cancer cells to specific sites of NETs accumulation thus reducing NETs-dependent metastatic dissemination.

## Supporting information

S1 Fig DNA degradation of NETs samples by DNAse 1 treatment. Samples of NETs-enriched suspension were incubated with DNAse 1 (10000 UI/ml) for 15 min and 30 min at room temperature and then loaded on 1.5% agarose gels (w/v). NETs DNA samples (8 μg) treated with DNAse 1 for 15 min (lane 3) or 30 min (lane 4) showed the same smearing pattern along the gel whereas the untreated NETs sample (1 μg) did not show the presence of DNA fragments and remained undigested at the loading site (lane 2). Lane 1 and 5 show DNA molecular weight markers (ladder base pairs).
(TIF)

S2 Fig Levels of α5, β1, αν and β3 single chain expression in K562 and K562ανβ3 cells by western blotting. Samples of whole cell lysates (40 μg of proteins) from K562 and K562ανβ3 cells were subjected to western blot analysis using anti-α5 (Chemicon), anti-β1 (Chemicon), anti-β3 (Santa Cruz) rabbit polyclonal antibodies and anti-αv (clone P2W7, Santa Cruz) mouse monoclonal antibody.
(TIF)

S3 Fig Expression of whole α5β1 and ανβ3 integrins in K562 and K562ανβ3 cells by FACS. Representative histograms from FACS analysis showing the percentage of K562 and K562ανβ3 cells expressing α5β1 and ανβ3 integrins as compared to control.
(TIF)

S4 Fig Western blot analysis of vitronectin expression. Samples of conditioned medium from unstimulated and stimulated dHL-60 or from cell-free NETs enriched suspension (50 μg of proteins) were subjected to western blot analysis using an anti-vitronectin monoclonal antibody (clone VIT-2, Sigma) and purified vitronectin (Promega) as positive control. Vitronectin was undetectable in all samples except positive control.
(TIF)

S1 Supporting Information (DOCX)
