# Supplementary figures and images for: Integrin-dependent cell adhesion to neutrophil extracellular traps through engagement of fibronectin in neutrophil-like cells
Source: PLoS One. 2017 Feb 6;12(2):e0171362. doi: 10.1371/journal.pone.0171362 (PMC5293257; doi:10.1371/journal.pone.0171362)

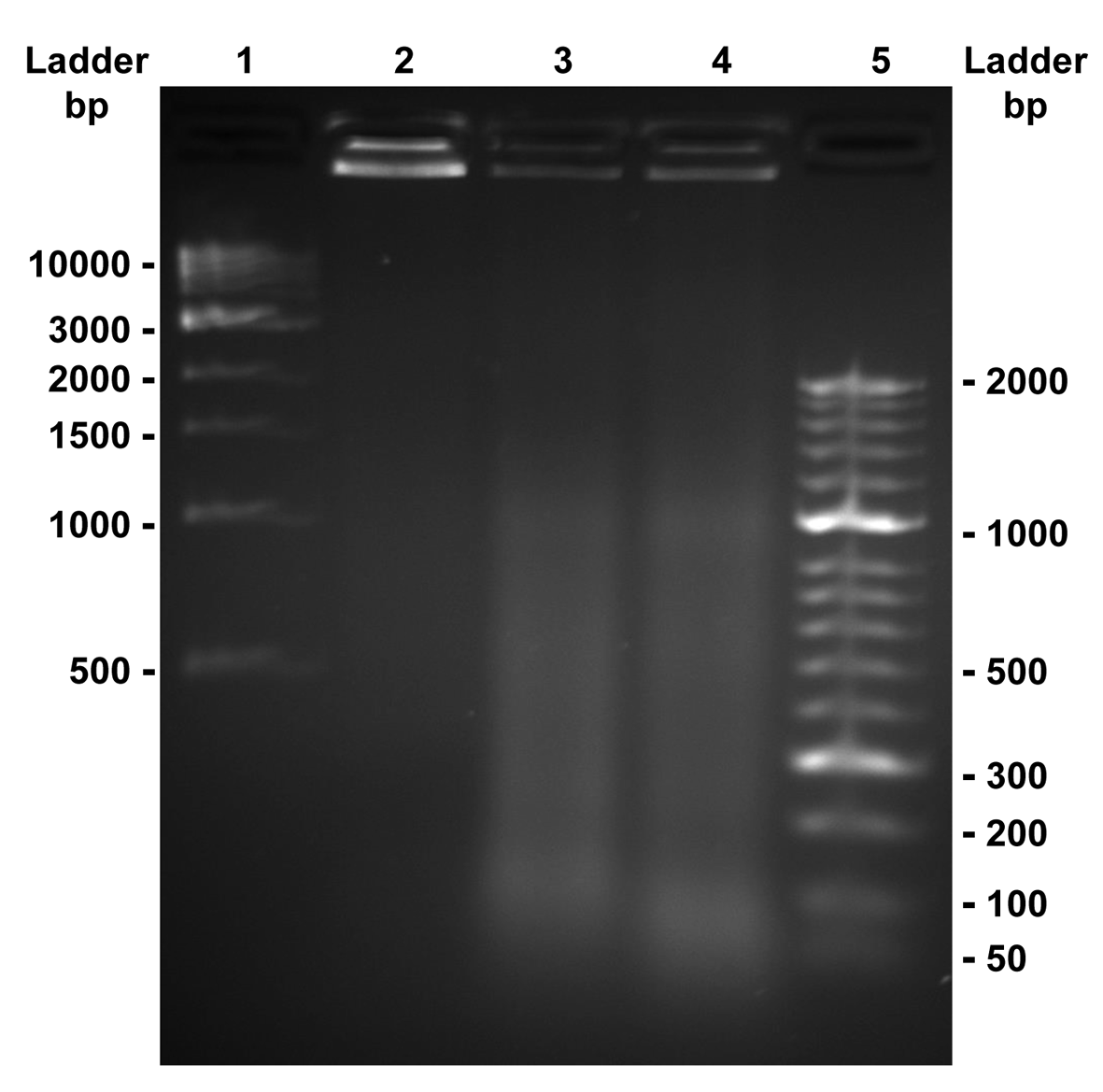

Supplement: S1 Fig — Samples of NETs-enriched suspension were incubated with DNAse 1 (10000 UI/ml) for 15 min and 30 min at room temperature and then loaded on 1.5% agarose gels (w/v). NETs DNA samples (8 μg) treated with DNAse 1 for 15 min (lane 3) or 30 min (lane 4) showed the same smearing pattern along the gel whereas the untreated NETs sample (1 μg) did not show the presence of DNA fragments and remained undigested at the loading site (lane 2). Lane 1 and 5 show DNA molecular weight markers (ladder base pairs). (TIF) [file pone.0171362.s001.tif]

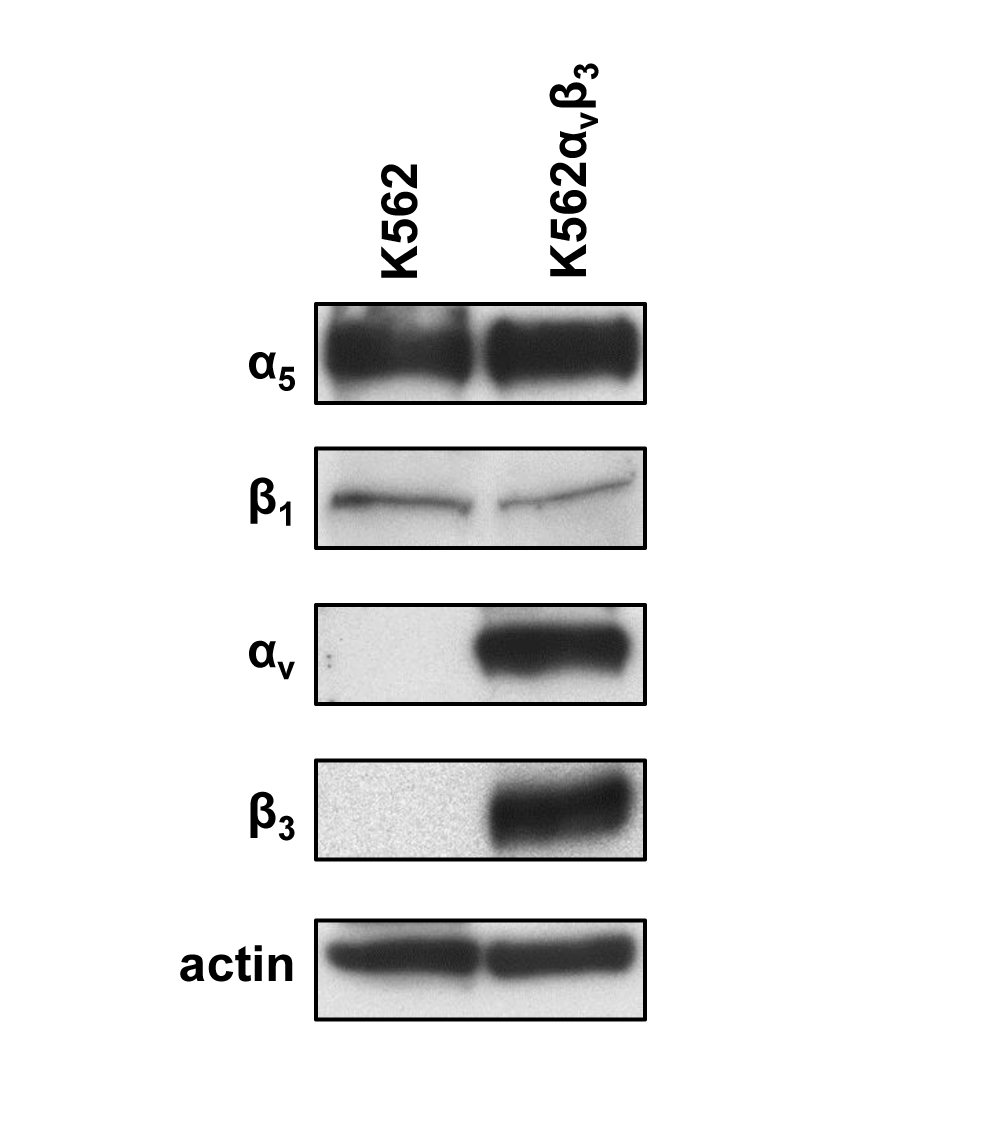

Supplement: S2 Fig — Samples of whole cell lysates (40 μg of proteins) from K562 and K562ανβ3 cells were subjected to western blot analysis using anti-α5 (Chemicon), anti-β1 (Chemicon), anti-β3 (Santa Cruz) rabbit polyclonal antibodies and anti-αv (clone P2W7, Santa Cruz) mouse monoclonal antibody. (TIF) [file pone.0171362.s002.tif]

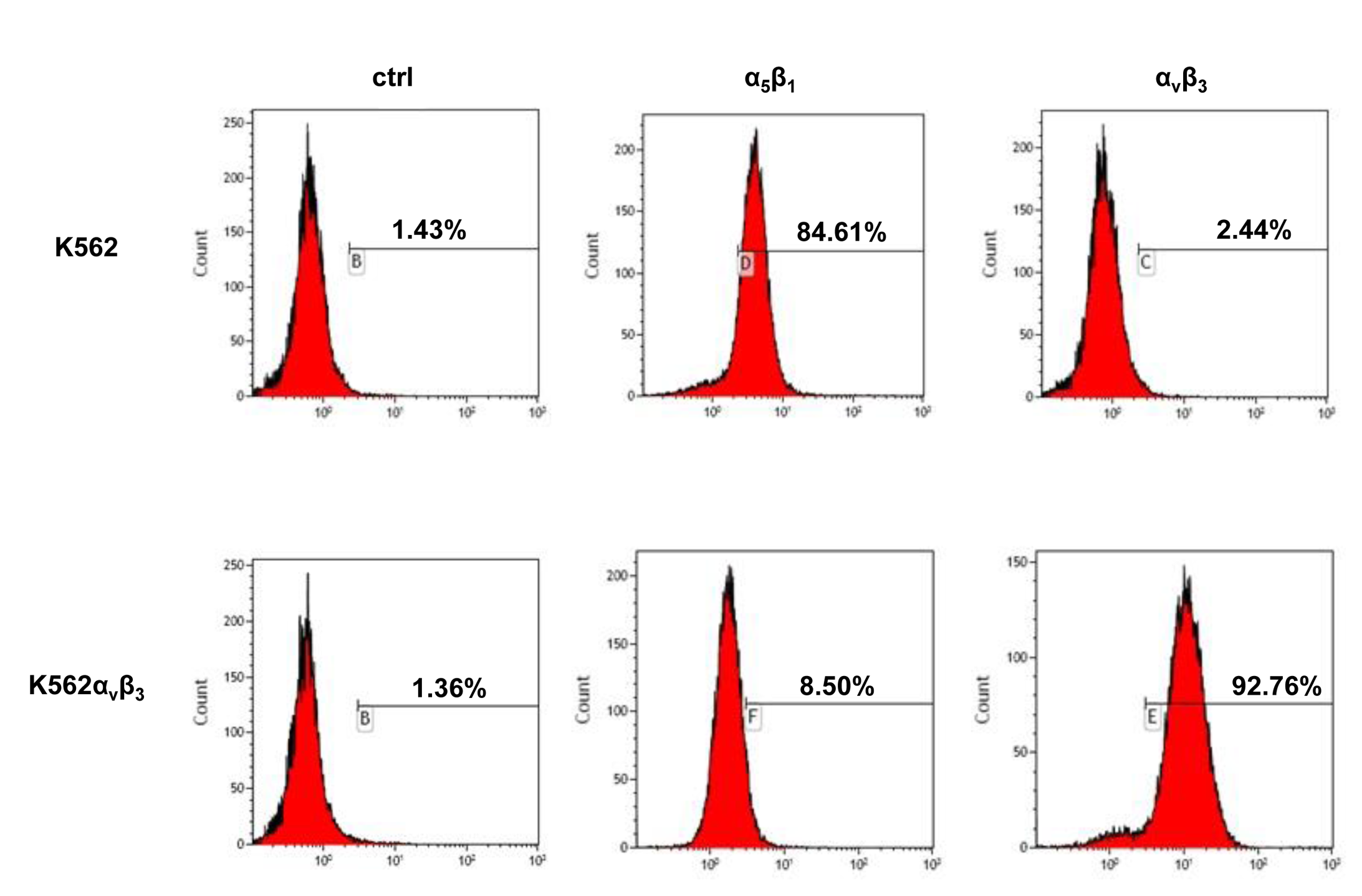

Supplement: S3 Fig — Representative histograms from FACS analysis showing the percentage of K562 and K562ανβ3 cells expressing α5β1 and ανβ3 integrins as compared to control. (TIF) [file pone.0171362.s003.tif]

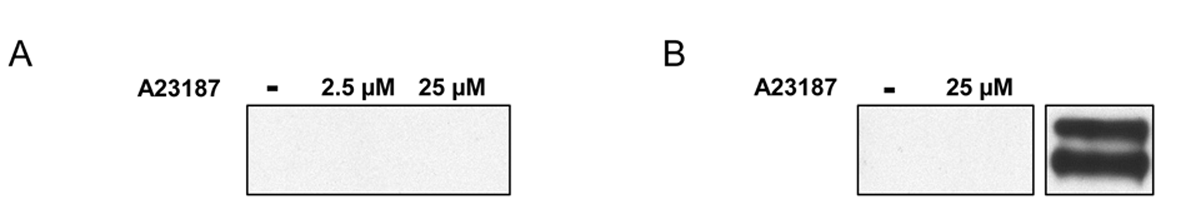

Supplement: S4 Fig — Samples of conditioned medium from unstimulated and stimulated dHL-60 or from cell-free NETs enriched suspension (50 μg of proteins) were subjected to western blot analysis using an anti-vitronectin monoclonal antibody (clone VIT-2, Sigma) and purified vitronectin (Promega) as positive control. Vitronectin was undetectable in all samples except positive control. (TIF) [file pone.0171362.s004.tif]
