# Supplementary material for: Integrin-dependent cell adhesion to neutrophil extracellular traps through engagement of fibronectin in neutrophil-like cells
Source: PLoS One. 2017 Feb 6;12(2):e0171362. doi: 10.1371/journal.pone.0171362 (PMC5293257; doi:10.1371/journal.pone.0171362)
Supplement: S1 Supporting Information — (DOCX) [file pone.0171362.s005.docx]

To confirm the optimal degradation of NETs samples in our study, they were incubated with DNAse 1 (10000 UI/ml) for 15 min and 30 min at room temperature and then loaded on 1.5 % agarose gels (w/v) prepared in Tris-borate-EDTA buffer containing 1 µg/ml ethidium bromide (Sigma). The results are shown in S1 Fig. Quantitative analysis of the agarose gel by ImageJ software (NIH, Bethesda, MD, USA) showed that the percentage of sample degradation at 15 min and 30 min was 95% and 93%, respectively, as compared to the untreated sample.
